# Ergonomic behaviour of learners in a digitally driven school environment: Modification using an ergonomic intervention programme

**DOI:** 10.4102/sajp.v74i1.348

**Published:** 2018-04-11

**Authors:** Ingrid V. Sellschop, Hellen Myezwa, Witness Mudzi, Eustatius Musenge

**Affiliations:** 1 Private, Johannesburg, South Africa; 2 Department of Physiotherapy, University of the Witwatersrand, South Africa; 3 Faculty of Health Sciences, University of the Witwatersrand, South Africa

## Abstract

**Background:**

Computer use is increasing amongst adolescents and so is the potential for related musculoskeletal pain and postural changes. The cumulative effect of this technology-induced, sedentary lifestyle leads to poor posture, pain, repetitive strain injury and dysfunctional movement patterns.

**Objectives:**

The purpose of this study was to establish the effect of a computer-related ergonomic intervention for adolescents in a school environment on posture and ergonomic behaviour.

**Methods:**

All Grade 8 learners at two randomly selected private schools in Johannesburg were invited to participate in the study (*n* = 127). A controlled trial compared an intervention group with a control group. The computer usage questionnaire and rapid upper limb assessment (RULA) were assessed at baseline, 3 and 6 months post-intervention. The intervention consisted of a participatory educational programme. An intention-to-treat analysis was undertaken. Alpha level was set at *p* = 0.05. Descriptive statistics (frequencies and percentages) and between-group analysis of variance, determined differences in the number of participants in the RULA action levels between groups after the intervention and the comparison of positions and type of computer.

**Results:**

At 6 months post-intervention, there were no participants in action level (AL) 4 and the number of participants in AL 3 had reduced from 26.2% at baseline to 14.8% in the intervention group (*p* < 0.001). The control group RULA scores worsened over the period of 6 months. Although the learners were still not in an ’acceptable’ range of postural positions, there was a significant improvement between the pre-intervention and post-intervention stage (*p* < 0.001).

**Conclusion:**

These findings demonstrate the effect of an ergonomic intervention and its sustainability over 6 months.

**Clinical implications:**

The clinical contribution of this study to our healthcare system is that through the early identification and intervention of the poor ergonomics in a school environment, a positive impact on reducing poor postural behaviour amongst learners can be achieved.

## Introduction

Life-skills education is important for promoting health and well-being amongst adolescents in a school environment. These skills comprise particular attitudes and knowledge, as well as skills that facilitate individuals to deal effectively with the challenges of physical, emotional and social well-being (Jacobs, Hudak & Mcgiffert 2003). Applied ergonomic skills related to computer use, posture and carrying schoolbags can play an important role in advocating change in the ergonomic behaviour amongst adolescents in a school environment, as well as creating awareness of healthy computing habits (Dockrell, Earle & Galvin 2010; Ismail et al. 2010; Jacobs et al. 2003).

Recent studies on the ergonomics of computer use by adolescents have investigated the potential effects of computer use on their health and productivity (Harris et al. 2015). The findings suggest that adolescents using computers may be at risk of developing musculoskeletal problems related to computer use (Harris & Straker 2000; Heyman & Dekel 2009). An increase in the time spent on a computer is significantly associated with increased reporting of musculoskeletal pain (Hakala et al. 2006; Katz 2006; Straker et al. 2006).

School-based ergonomic intervention studies between 2009 and 2011 have all made use of an educational ergonomic programme approach with applied ergonomic principles, stretches, posture education and demonstrations thus following the principles and concepts of learning theory and behaviour modification. Ergonomic interventions that educate adolescents about posture, applied ergonomic principles and stretch exercises make a significant difference to the prevalence of musculoskeletal pain (Dockrell et al. 2010; Heyman & Dekel 2009; Robbins, Johnson & Cunliffe 2009; Sawyer & Penman 2011). All studies cited were conducted in different geographical areas, indicating that the importance of ergonomics in the school environment is a worldwide concern (Ireland, the United States, Malaysia, Israel and Australia).

Linton et al. (1994) and Jeffries, Milanese and Grimmer-Somers (2007) have shown that merely adapting school furniture has, on its own, proved to be neither viable nor sustainable for preventing back pain in adolescents as one needs to include postural education. To get better results in facilitating a change in ergonomic behaviour and posture amongst adolescents and to prevent musculoskeletal pain, it is important to assess the posture of adolescents in relation to computer use and workstation design in a school environment. Following an assessment of these components, the literature supports the design of an effective ergonomic intervention with the inclusion of an educational component comprising aspects of applied ergonomic principles, the correct use of stretch exercises and ‘pause’ breaks when using a computer as well as teaching adolescents about the importance of being active in between computer use (Dockrell et al. 2010; Ismail et al. 2010).

At present, there have been no longitudinal ergonomic intervention studies done in Africa and there is certainly evidence in the literature that indicates that computer use in children in African countries, in particular South Africa, is on the increase (Brink et al. 2015; Van Niekerk et al. 2013). In addition, evidence pertaining to longitudinal randomised control trials of ergonomic interventions in schools and their effect on key outcomes such as posture and musculoskeletal pain is scarce. The majority of the studies (70%) reviewed were quasi-experimental and only three prospective longitudinal studies by Saarni et al. (2009), Jacobs et al. (2003) and Dolphens et al. (2011) had been conducted at the time of this study.

Thus, the purpose of this study was to establish the effect of a computer-related ergonomic intervention for adolescents in a school environment, on posture and ergonomic behaviour.

## Method

A single blind control trial was conducted with assessments at baseline, 3 and 6 months after the intervention. Two schools were chosen using randomised cluster sampling from a population of 27 co-educational private secondary schools in the greater Johannesburg region.

The intervention and control groups were assessed by the first author and research assistant at baseline prior to the intervention and then again at 3 and 6 months after the start of the intervention. They were blinded to group allocation and to the delivery of the ergonomic intervention programme to the participants to limit assessment bias and knowledge of which school received the intervention. The participants were assessed in a venue separate from the intervention classroom to assure blinding of the first author and research assistant.

Both the intervention and the control groups completed the computer usage questionnaire (CUQ) (Smith et al. 2007) at baseline, 3- and 6-month intervals post-intervention. The CUQ was developed by Smith et al. (2007) and has been tested for content and face validity and reliability (stability) by Smith et al. (2007). For the purposes of this study, the intra-rater reliability of the CUQ was tested in a pilot study and found to have good reliability (ICC = 0.99, *p* < 0.01).

All participants underwent biometric measurements of height, weight and school bag weight and postural analysis using the validated rapid upper limb assessment (RULA) (McAtamney & Corlett 1993). The RULA postural survey method was developed for use in ergonomic investigations; scores are combined and the observed posture assigned to an action level (AL) which indicates the required intervention (McAtamney & Corlett 1993). An AL 1 is the optimal postural position and an AL 4 is a high-risk postural position, which requires intervention immediately. The RULA is a valid tool (McAtamney & Corlett 1993), with good inter-rater reliability (*r* = 0.77, *p* < 0.001) (Laeser, Maxwell & Hedge 1998). Participants in both groups had their postures assessed for 1 min each with RULA, while they were using a computer at baseline, 3 and 6 months.

The intervention group received a one-off 45-min participative intervention programme delivered by a university physiotherapy lecturer, who had been trained in the programme delivery, 2 weeks after baseline data were collected by the first author and the research assistant. The programme comprised an educational ergonomics component on posture, bag weight (10% of body weight) and workstation set-up as well as a component of stretches for the neck, shoulders and lower back. The format included a Power Point presentation with planned activities for the participants. The computer-related ergonomic intervention programme was developed from the few intervention studies that have been done (Heyman & Dekel 2009; Ismail et al. 2010; Robbins et al. 2009) and was evaluated by four educators, eight adolescents and an expert in the field of ergonomics during a pilot study, and modified according to suggestions made. A poster demonstrating correct workstation set-up and a variety of stretches was placed in the computer classroom of the intervention group. Thereafter, each participant was given a sticker to place on his or her screen at home and at school. This sticker, a red dot, acted as a reminder to the participants to adjust their posture and to do their stretches during the time that they spent on the computer. A free web-based link (http://blogs.bu.edu/kjacobs/) was given to each participant to download onto their home computer to reinforce the reminder of doing stretches and taking regular short breaks from computer use when at home. All participants were given a short multiple choice questionnaire test immediately after the intervention, to test their comprehension and understanding of the ergonomic concepts that they had been taught.

The control group participants were not exposed to any ergonomic intervention programme as they were in a different school from the intervention group.

An intention-to-treat analysis was undertaken (Hollis & Campbell 1999). Results were analysed using SPSS 20.0.0 for Windows. The alpha level was set at *p* = 0.05. Descriptive statistics (frequencies and percentages) and between-group ANOVAs were used to summarise RULA action levels to determine differences in the number of participants in the RULA action levels between the control and intervention groups and the comparison of positions and type of computer use over the study period. Repeated measures ANOVA were used to measure the effect of the intervention and compare changes between groups for the RULA wrist/arm and neck/trunk/leg scores. The within group analysis was done using a Stuart–Maxwell test. As the data were not normally distributed at baseline, propensity score matching of the RULA scores was done accounting for differences between the groups at baseline. A generalised estimated equations (GEE) model was used to estimate the average response of the RULA scores over the population.

### Ethical considerations

Ethical clearance was granted by the Human Research Ethics Committee (HREC) of the University of the Witwatersrand for the study (M110128). Permission was obtained from the head of the Independent School Association and the principals of the schools to conduct the study. Children who signed assent and whose parents signed informed consent were included. School A (*n* = 66) and school B (*n* = 61) were allocated to either the control or the intervention group.

## Results

**FIGURE 1:**
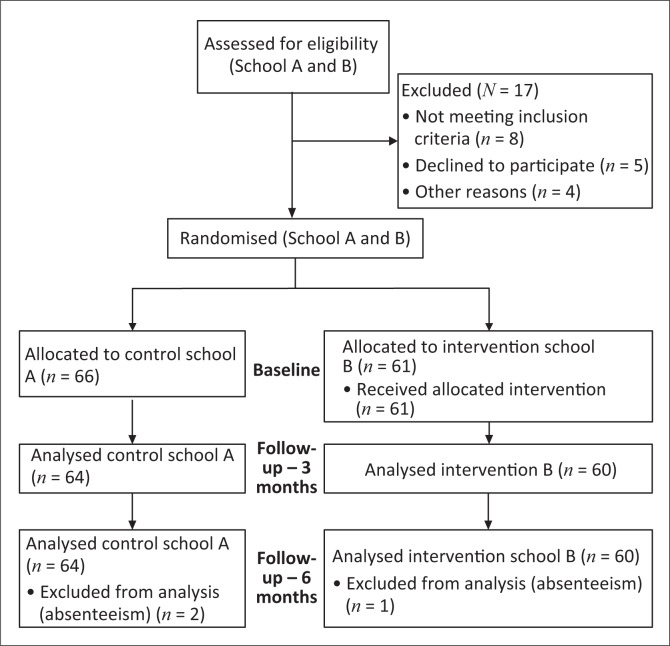
Consort diagram showing the flow of participants in the randomised control trial study.

Figure 1 illustrates the flow of participants through the study.

There were no significant differences between the control and intervention groups at baseline with respect to age and gender (Table 1).

**TABLE 1:** Demographic characteristics of the learners (*n* = 127).

Variables	Control	Intervention	*p*	Total
**Gender, *n* (%)**				
Male	39(59)	37(61)	*p* > 0.05	76(60)
Female	27(41)	24(39)		51(40)
**Age, mean (± SD)**				
Male	13.5(± 0.6)	13.5(± 0.7)	*p* > 0.05	13.5(± 0.6)
Female	13.2(± 0.4)	13.3(± 0.6)		13.3(± 0.5)
Overall age	13.4(± 0.5)	13.4(± 0.7)		13.3(± 0.6)

Most participants in the control and intervention groups used desktop computers rather than laptops. In the control and the intervention groups, 10 (15%) and 25 (41%) of the participants used laptops, respectively. More participants in the intervention group (7, 6.5%) used a laptop on the floor than the control group (2, 4%) at baseline. This changed significantly (*p* = 0.04) after 6 months with 14 (21%) of the participants in the control group using a laptop on the floor compared with 3 (5%) in the intervention group (Table 2).

**TABLE 2:** Positions and type of computer usage at baseline, 3 months and over a period of 6 months (*n* = 127).

Variables	Control Group (*n* = 66) *n* (%)	Intervention Group (*n* = 61) *n* (%)	*p*
**Type of computer use (baseline)**			
Desktop	54 (82)	32 (52)	0.02
Laptop	10 (15)	25 (41)	0.04
Laptop on floor	2 (4)	7 (6.5)	0.14
**Type of computer use (3 months)**			
Desktop	51 (78)	48 (79)	0.51
Laptop	12 (19)	9 (15)	0.58
Laptop on floor	3 (4.5)	4 (7)	0.63
**Type of computer use (6 months)**			
Desktop	45 (68)	42 (69)	0.51
Laptop	8 (12)	16 (26)	0.05
Laptop on floor	14 (21)	3 (5)	0.04

The mean weight of the schoolbags of the participants in the control group was reduced from 6.8 (± 2.5) kg to 6.7 (± 6.0) kg (*p* = 0.04) from baseline to 6 months while that for the intervention group reduced from 7.1 (± 2.0) kg to 5.4 (± 3.6) kg (*p* = 0.02).

At baseline, there were more participants in RULA A1 and A2 in the intervention group than in the control group (*p* = 0.05), and at 6 months, there were more learners in AL 1 and AL 2 in the intervention group (*p* = 0.001) than in the control group (Table 3). There were no differences in the RULA subscales between the groups after the intervention.

**TABLE 3:** A comparison of the number of participants in rapid upper limb assessment action levels between the control and intervention groups at baseline, 3 months and 6 months (*n* = 127).

Variables	Control group (*n* = 66) *n* (%)	Intervention group (*n* = 61) *n* (%)	*p*
Action level 1	0 (0.0)	6 (4.7)	
Action level 2	27 (40.9)	26 (42.6)	
Action level 3	15 (22.7)	14 (21.3)	0.050
Action level 4	24 (36.4)	20 (26.2)	
Action level 1	12 (18.1)	6 (9.8)	
Action level 2	39 (59.1)	44 (72.1)	
Action level 3	14 (21.1)	10 (18.0)	0.300
Action level 4	1 (1.5)	0 (0.0)	
Action level 1	5 (7.6)	18 (29.5)	
Action level 2	22 (31.8)	35 (55.7)	
Action level 3	19 (28.8)	9 (14.8)	0.01
Action level 4	22 (31.8)	0 (0.0)	

The results in Table 4 show that there was a significant improvement in the wrist/arm (RULA) scores of the intervention group at 6 months (confidence interval [CI]: 3.08–4.07, *p* < 0.001) and in the neck/trunk/leg score (CI: 2.53–4.41, *p* = 0.001) relative to the control group.

**TABLE 4:** Rapid upper limb assessment scores between the control and intervention group at baseline, 3 months and over a period of 6 months (*n* = 127).

Variables	Control	Intervention	95% CI	*p*
	Mean (± SD)	Mean (± SD)		
**RULA final wrist/arm scores**				
Baseline	5.1 (± 0.9)	4.5 (± 1.7)	4.36–5.08	0.800
3 months	3.4 (± 1.7)	3.7 (± 1.3)	3.33–4.06	0.400
6 months	4.4 (± 1.4)	3.0 (± 2.0)	3.08–4.07	0.001
**RULA neck, trunk and leg scores**				
Baseline	4.4 (± 1.1)	4.1 (± 2.2)	3.34–5.30	0.800
3 months	2.1 (± 1.9)	2.1 (± 1.5)	3.79–3.64	0.100
6 months	4.5 (± 2.3)	2.2 (± 1.8)	2.53–4.41	0.001

RULA, rapid upper limb assessment; CI, confidential interval; SD, standard deviation.

Table 4 shows the results from the propensity score matching of the RULA final neck/trunk/leg scores using a two-sample *t*-test. The results showed that there was only a significant difference in the RULA final neck/trunk/leg scores (CI 2.53–4.41, *p* = 0.001) after 6 months with the new adjusted sample which is a similar trend found in the sample obtained from the cluster randomised sampling (Table 4) and thus the selection bias did not have an effect on the intervention results.

A GEE model was used to estimate the average response of the RULA final wrist/arm scores and the RULA final neck/trunk/leg scores over the population. An auto-aggressive first-order correlation structure was used and the GEE found that the way the learners were at baseline, based on their age, gender and weight influenced the way they were at 3 months and in turn this influenced their outcome at 6 months in terms of their wrist and arm position while using a computer (Table 5). The GEE results are shown to support the design used in this study as the participants who were heavier were found to be at a greater risk for developing poor wrist and arm positions while using the computer.

**TABLE 5:** Generalised estimated equations model for rapid upper limb assessment.

Variables	OR	SE	SD	*p*	CI
**RULA wrist/arm scores**					
Age	1.0	0.040	0.7	0.5	0.94–1.12
Gender (female)	1.0	0.050	0.8	0.4	0.94–1.15
Weight	0.1	0.002	−0.6	0.6	0.14–1.00
**Total-PCS**	**1.0**	**0.003**	**0.5**	**0.6**	**0.15–1.00**
**RULA neck/trunk/leg scores**					
Age	1.0	0.050	0.9	0.4	0.94–1.15
Gender (female)	1.0	0.060	1.3	0.2	0.96–1.22
Weight	0.1	0.002	−1.45	0.1	0.99–1.00
**Total-PCS**	**0.1**	**0.003**	**−0.28**	**0.8**	**0.99–1.00**

OR, odds ratio; SE, standard error; SD, standard deviation; PCS, pain catastrophising score; CI, confidence interval; RULA, rapid upper limb assessment.

There was a significant difference in scores within the intervention group between baseline and 6 months (*p* < 0.011), indicating that the posture of the learners relating to their neck, trunk and leg positions had improved over a period of 6 months but no difference was found in the control group (Table 6).

**TABLE 6:** Rapid upper limb assessment scores within groups over the study period.

Variables	Control			Intervention		
	Mean diff.	95% CI	*p*	Mean diff.	95% CI	*p*
**RULA wrist/arm scores**						
Baseline–3 months	1.70	(1.23 to 2.17)	0.00 1	0.8	(0.42–1.18)	0.001
3 months–6 months	−1.03	(−2.17 to −1.23)	0.001	1.5	(1.75–2.17)	0.020
Baseline–6 months	0.70	(0.26 to 1.07)	0.002	0.7	(0.10–1.22)	0.001
**RULA neck/trunk/leg scores**						
Baseline–3 months	1.50	(0.78 to 2.13)	0.001	1.2	(0.62–1.68)	0.001
3 months–6 months	−1.50	(−2.19 to −0.81)	0.001	0.8	(0.24–1.40)	0.006
Baseline–6 months	−0.05	(−0.78 to 0.69)	0.900	1.1	(1.23–2.71)	0.001

RULA, rapid upper limb assessment; CI, confidential interval.

## Discussion

The main findings of this study were that a change in behaviour in terms of positioning and body mechanics was observed in the intervention group, suggesting that the 45 min participatory ergonomic intervention programme was effective. The position of the computer while being used is an important predictor for developing musculoskeletal pain as this relates to the concept of ergonomic behaviour. In this study, participants’ ergonomic behaviour was assessed by asking them to indicate where their computer was positioned when using it outside of school, for example ‘on their lap’ or ‘on the floor’. Working on a laptop on the floor puts the participant in a less favourable postural position of trunk and neck flexion with hyperextension of the upper cervical spine (Straker et al. 2006). After 6 months, there was a significant reduction in the number of participants who used a laptop on the floor in the intervention group compared with participants in the control group, and this could have been because of the educational input.

A previous study presenting a cognitively based, 50-min body mechanics education programme to 141 students in Grades 1 to 6 reported similar results (Schwartz & Jacobs 1992). They asserted that although long-term learning is essential for changes in behaviour to occur, the efficacy of the educational programme is best measured by a change in performance, for example, postural positioning and interaction with different forms of IT, rather than knowledge retention. Our findings relating to the intervention group support the implementation of affordable and appropriately developed interventions. In terms of altering behaviour in adolescents in a school environment, it is essential to consider the influence of their social environment at school and in the home, as well as their attitude towards pain in general (Dolphens et al. 2011; Dunn et al. 2011). Hence, implementing the intervention programme in the school environment and sending the participants home with tools for reinforcing concepts learnt from the intervention support the theoretical underpinnings of cognitive behavioural change (Mennuti et al. 2012)

When the intervention and control groups were compared for RULA action levels, compared with baseline, the control group presented with a worse picture at 6 months where 29% and 32% were in AL 3 and AL 4, respectively, compared with there being no learners at AL 4 in the intervention group. The study by Dockrell et al. (2010) on the effects of a school ergonomic intervention involving children using computers had similar results. In our study, the majority of participants at the pre-intervention stage were in AL 3 and only 10% were in AL 2. Post-intervention, there was a significant shift in the RULA scores such that 91% had shifted to AL 2. This is similar to Syazwan et al.’s (2011) study, where participants in the intervention group (*n* = 78) were found to have shifted from AL 4 to AL 3, while those in the control group worsened in posture at the follow-up assessment. The benefits of maintaining a good posture early on in childhood and adolescence, and being aware of the effects of sedentary sitting behaviour when working on a computer have been well documented (Cardon et al. 2004; Grimmer & Williams 2000; Straker, Briggs & Greig 2002).

There was a significant improvement (*p* = 0.03) between the final wrist/arm scores (RULA) at 6 months (*p* < 0.01) post-intervention in the intervention group compared with the control group. Therefore, the ergonomic intervention seems to have had an effect on the learners’ behaviour with regard to their wrist/arm positioning when using a computer. This means that the participants seem to have responded favourably to the intervention programme and a change in behaviour was observed over time, suggesting that ergonomic interventions in schools can reduce the risk factor of poor posture, resulting in perpetual musculoskeletal pain. Dockrell et al. (2010) reported similar findings where the upper limb RULA score (mean wrist/arm RULA score) pre-intervention was 4.8 and 3.8 post-intervention (*p* < 0.01), thus reducing poor posture as an ergonomic risk factor amongst school children using computers.

Similarly, a significant improvement for neck/trunk/leg RULA scores was found at 6 months post-intervention (*p* < 0.01), indicating that the intervention may have had an effect on the upper body and neck position of the intervention group. Again, the findings are similar to the reduction in the neck/trunk/leg RULA scores post-intervention in the study by Dockrell et al. (2010). They found a significant reduction in the mean RULA scores pre-intervention (mean = 5.7) compared with the mean RULA score (mean = 3.9) post-intervention.

These findings of RULA action levels and RULA scores (final arm/wrist and neck/trunk/leg) support an interpretation that the intervention had a positive and sustained effect on the posture of participants over the 6-month period as their posture improved and none were found to be in a high-risk postural position (AL 4) at 6 months post-intervention. The shift to a more acceptable action level suggests that the reinforcements from the intervention used during the course of the 6 months assisted in facilitating a change in the postural activity of the learners and potentially reduced the ergonomic risk for developing musculoskeletal pain when using a computer.

The ergonomic intervention studies by Dockrell et al. (2010), Syazwan et al. (2011) and Ismail et al. (2010) all implemented a similar type of educational ergonomic intervention programme. They also used visual and graphic aids, problem-solving strategies for adjusting workstations and stretch exercises. The results reported are similar to this study for participants in a school environment in that they reported better RULA measurements (Breen et al. 2007; Dockrell et al. 2010; Ismail et al. 2010; Oates, Evans & Hedge 1998; Syazwan et al. 2011).

Our study is the first longitudinal study to consider the effects of a computer-related ergonomic intervention on posture and ergonomic behaviour in a school environment in South Africa. It gives insight into the probability of the impact such an intervention can have and whether the effects are sustainable in a digitally driven school environment.

Limitations of this study relate to the sample which represented only Grade 8 adolescents from high fee-paying schools in the Johannesburg area. A further limitation of this study was that it considered only computer-related ergonomics as a risk factor for musculoskeletal pain.

## Conclusion

The main findings of this study were that a change of behaviour in terms of positioning and body mechanics was observed in the intervention group, suggesting that the 45-min participatory ergonomic intervention programme was effective.

An ergonomic intervention programme in a school environment can be effective in improving posture and the effect can be sustained over a period of 6 months.
